# Increased Risk of Ischemic Stroke in Young Patients with Ankylosing Spondylitis: A Population-Based Longitudinal Follow-Up Study

**DOI:** 10.1371/journal.pone.0094027

**Published:** 2014-04-08

**Authors:** Chia-Wei Lin, Ya-Ping Huang, Yueh-Hsia Chiu, Yu-Tsun Ho, Shin-Liang Pan

**Affiliations:** 1 Department of Physical Medicine and Rehabilitation, National Taiwan University Hospital, Taipei, Taiwan; 2 Department of Physical Medicine and Rehabilitation, National Taiwan University Hospital Yu-Lin Branch, Yunlin, Taiwan; 3 Department and Graduate Institute of Health Care Management, Chang Gung University, Tao-Yuan, Taiwan; 4 Department of Physical Medicine and Rehabilitation, National Taiwan University College of Medicine, Taipei, Taiwan; Oregon Health & Science University, United States of America

## Abstract

**Background:**

Prospective data on the association between ischemic stroke and ankylosing spondylitis (AS) in the young are sparse. The purpose of this population-based, age- and sex-matched longitudinal follow-up study was to investigate the risk of developing ischemic stroke in young patients with AS.

**Methods:**

A total of 4562 patients aged 18- to 45-year-old with at least two ambulatory visits in 2001 with a principal diagnosis of AS were enrolled in the AS group. The non-AS group consisted of 22810 age- and sex-matched, randomly sampled subjects without AS. The two-year ischemic stroke-free survival rate for each group were calculated using the Kaplan-Meier method. Cox proportional hazards regression analysis was used to estimate the hazard ratio of ischemic stroke after adjusting for demographic and clinical covariates.

**Results:**

During follow-up, 21 patients in the AS group and 53 in the non-AS group developed ischemic stroke. The ischemic stroke-free survival rate over the 2 year follow-up was lower in the AS group than the non-AS group (p = 0.0021). The crude hazard ratio of ischemic stroke for the AS group was 1.98 (95% CI, 1.20–3.29; p = 0.0079) and the adjusted hazard ratio after controlling for demographic and comorbid medical disorders was 1.93 (95% CI, 1.16–3.20; p = 0.0110).

**Conclusion:**

Our study showed an increased risk of developing ischemic stroke in young patients with AS.

## Introduction

Ankylosing spondylitis (AS), an autoimmune disease with systemic inflammation, predominantly involves the axial skeleton [1]. AS has been associated with an increased risk of ischemic heart disease [2]–[8], but whether AS patients are at a higher risk of ischemic stroke remains controversial. Two observational studies have reported an increased risk of cerebrovascular disease in AS patients [9], [10], whereas another study found that AS patients had no increased prevalence of stroke compared to non-AS patients [11]. In addition, two of these studies were based on a cross-sectional survey [9], [11] and prospective data on the relationship between AS and cerebrovascular diseases are sparse. Moreover, these studies were carried out mainly on middle-aged or older AS patients, and little is known about cerebrovascular risk in young AS patients. Thus, the aim of this population-based, age- and sex-matched longitudinal follow-up study was to evaluate the risk of developing ischemic stroke in young patients with AS.

## Materials and Methods

### Data source

The data used in this study were obtained from the complete National Health Insurance (NHI) claim database in Taiwan for the period 2000 to 2003. The NHI program, a single-payer compulsory social insurance program, has been implemented in Taiwan since 1995, and the coverage rate was 96% of the whole population in 2000 and 97% at the end of 2003, at which time more than 21.9 million inhabitants were enrolled. It should be noted that the rationale for using the NHI database after 2000 is that, from Jan 1st, 2000, according to the rules of the Bureau of NHI, NHI claim data have been encoded using the standardized International Classification of Disease, 9^th^ Revision, Clinical Modification (ICD-9-CM).

### Ethics Statement

To keep individual information confidential so as to satisfy regulations on personal privacy in Taiwan, all personal identification numbers in the data were encrypted by converting them into scrambled numbers before data processing. This study was exempt from full review by the National Taiwan University Hospital Research Ethics Committee, and the need for informed consent was waived because the data used consisted of de-identified secondary data released for research purposes and were analyzed anonymously, thus complying with the regulations of the Department of Health, Executive Yuan, Republic of China.

### Study Design and Subjects

We used an age- and sex-matched cohort study design to investigate the risk of ischemic stroke in young patients with AS. The study population included an AS group and a non-AS group. The AS group consisted of subjects aged between 18 to 45 years who had received a principal diagnosis of AS (ICD-9-CM code 720.0) in ambulatory medical care visits between January 1, 2001 and December 31, 2001. The index visit was defined as the first ambulatory visit during which the principal diagnosis of AS was made. In order to maximize case ascertainment, only patients with at least two ambulatory visits (including the index visit) with a principal diagnosis of AS in this period were considered for inclusion in the AS group (n = 11428). The exclusion criteria for the recruitment of subjects into the AS group were: (1) a previous diagnosis of AS during year 2000 (n = 6473) to increase the likelihood of identifying AS cases newly diagnosed in 2001; (2) a previous diagnosis of any type of stroke (ICD-9-CM codes 430-438) (n = 91) before the index visit; and (3) a diagnosis of diffuse diseases of connective tissue (ICD-9-CM code 710, n = 252) or rheumatoid arthritis (ICD-9-CM code 714, n = 765) before the index visit; 6866 subjects were excluded because of one or more of these criteria, leaving 4562 subjects in the final AS group.

The non-AS group was constructed by sampling the subjects without a diagnosis of AS in the same 2001 NHI claim database. We assigned the first ambulatory visit during 2001 as the index visit. The exclusion criteria for recruiting subjects into the non-AS group were: (1) a previous diagnosis of AS before the index visit; (2) a previous diagnosis of stroke before the index visit; and (3) a diagnosis of diffuse diseases of connective tissue or rheumatoid arthritis before the index visit. We randomly sampled 5 age- and sex-matched subjects for each subject in the AS group. A total of 22810 subjects was included in the non-AS group.

### Outcome

All ambulatory medical care and inpatient records for each subject in the two groups for the 2 year follow-up period were retrieved and the mortality data for those subjects who died during follow-up were obtained from the mortality registry. These medical records and mortality data were linked through a unique encrypted identification number for each subject. We identified the date of the first principal diagnosis of ischemic stroke (ICD-9-CM codes 433–437) during follow-up as the primary endpoint. All subjects were followed from the index visit to the first occurrence of ischemic stroke, death, or end of follow-up (whichever occurred first). We evaluated the effect of AS on the ischemic stroke-free survival, adjusting for the demographic features of age and sex and for the cardiovascular comorbidities of hypertension (ICD-9-CM code 401–405), diabetes (ICD-9-CM code 250), dyslipidemia (ICD-9-CM code 272), coronary heart disease (ICD-9-CM codes 410–414, and 429.2), and other types of heart disease (ICD-9-CM code 393-398, 420–429). Information on these preexisting comorbid medical disorders was obtained by retrieving all the ambulatory medical care and inpatients records in the NHI database for the year before the index visit.

### Statistical analysis

The Chi-square test and Student's *t* test were used to compare differences in demographic characteristics and comorbid medical disorders between the AS and non-AS groups. The ischemic stroke-free survival probabilities for the two groups were estimated using the Kaplan-Meier method and differences in ischemic stroke-free survival between the two groups were tested using the log rank test. Cox proportional hazards regression analysis was used to estimate the effect of AS on the subsequent occurrence of ischemic stroke after adjusting for medical comorbidities. Univariate analysis was initially performed for each variable, then the best subset selection method was used to obtain the final multiple regression model. An alpha level of 0.05 was considered statistically significant. The analyses were performed using SAS 9.2 software (SAS Institute, Cary, NC).

## Results

Table 1 shows the distribution of demographic characteristics and medical comorbidities in the AS and non-AS groups. Men made up 73.8% of the AS group, which had a mean age of 31.3 years (SD  = 7.6). The AS group had a higher prevalence of dyslipidemia (p = 0.0157), coronary heart disease (p<0.0001), and other types of heart disease (p<0.0001). There was no significant difference between the two groups in the prevalence of diabetes mellitus (p = 0.3755) or hypertension (p = 0.7527).

**Table 1:** Demographic and clinical characteristics of the AS and non-AS groups.

Variable	AS group	Non-AS group	p value
	N = 4562	N = 22810	
Men	3365 (73.8)	16825 (73.8)	1.0000
Age (years)	31.3±7.6	31.2±7.6	0.2866
Diabetes	72 (1.6)	321 (1.4)	0.3755
Hypertension	102 (2.2)	493 (2.2)	0.7527
Dyslipidemia	122 (2.7)	479 (2.1)	0.0157
Coronary heart disease	61 (1.3)	171 (0.8)	<0.0001
Other heart diseases	122 (2.7)	285 (1.2)	<0.0001

Values are expressed as the mean ± SD or n (%).

**Figure 1:**
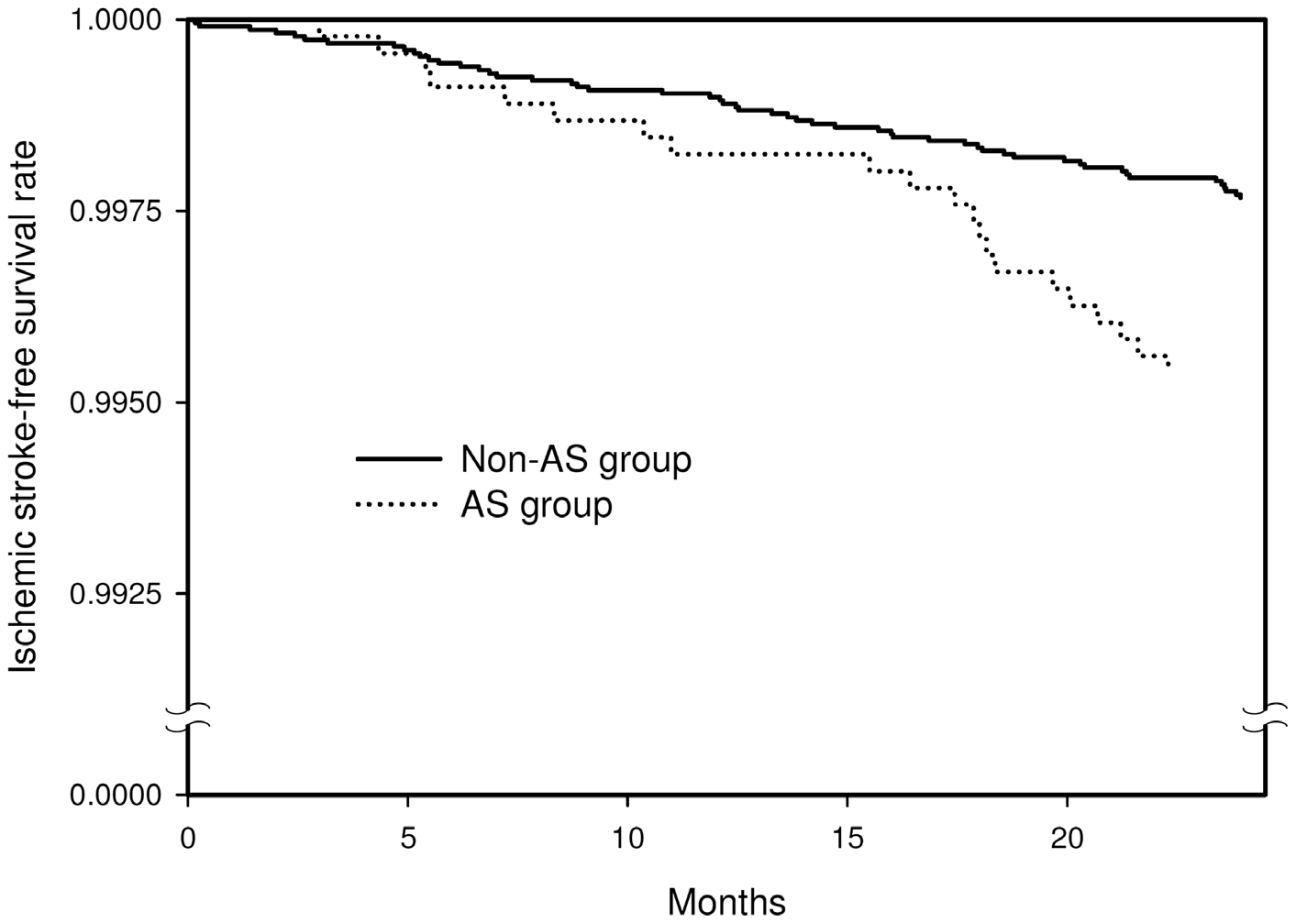
Two-year ischemic stroke-free survival rates for the ankylosing spondylitis (AS) group (dotted line) and non-AS group (solid line).

Of the 4562 subjects with AS, 21 (0.46%) developed ischemic stroke during the 2-year follow-up compared to 53 (0.23%) of the 22810 subjects in the non-AS group. Comparison of the ischemic stroke-free survival curves showed that the stroke-free survival rate for the AS group was significantly lower than that for the non-AS group (p = 0.0021, Figure 1).

The results of the Cox proportional hazards regression analysis are shown in Table 2. The left column shows the crude hazard ratio (HR) for each variable based on the univariate analysis. The covariates with a p value less than 0.05 were age, AS, hypertension, coronary heart disease, and other heart diseases. Compared to the non-AS group, the crude HR of ischemic stroke for the AS group was 1.98 (95% CI, 1.20 to 3.29; p = 0.0079). The middle column shows the results using the full multivariate model; age and sex were not included in the multiple regression analysis, since the AS and non-AS groups were matched for these variables. The right column shows the result of the final best subset selected model; the variables included in the final model were AS, hypertension, and coronary heart disease. The adjusted HR of developing ischemic stroke during the 2-year follow-up was 1.93 (95% CI, 1.16 to 3.20; p = 0.0110) for the AS group compared to the non-AS group. It should also be noted that hypertension and coronary heart disease were also associated with a higher risk of ischemic stroke.

**Table 2:** Crude and adjusted hazard ratio (HR) for the occurrence of ischemic stroke during the two-year follow-up period in the AS and non-AS groups.

	Occurrence of ischemic stroke					
	Univariate analysis		Full multivariate model		Best subset selected model	
Variable	Crude HR (95% CI)	p value	Adjusted HR (95% CI)	p value	Adjusted HR (95% CI)	p value
Age (years)	1.12 (1.08 to 1.16)	<0.0001	NA	NA	NA	NA
Sex (female vs. male)	0.97 (0.58 to 1.63)	0.9060	NA	NA	NA	NA
AS (AS vs. non-AS)	1.98 (1.20 to 3.29)	0.0079	1.92 (1.16 to 3.19)	0.0116	1.93 (1.16 to 3.20)	0.0110
Hypertension (yes vs. no)	6.32 (3.18 to 12.69)	<0.0001	4.55 (2.02 to 10.23)	0.0002	4.66 (2.15 to 10.09)	<0.0001
Diabetes mellitus (yes vs. no)	0.96 (0.13 to 6.86)	0.9633	0.42 (0.06 to 3.19)	0.4031	NA	NA
Dyslipidemia (yes vs. no)	2.56 (0.93 to 7.01)	0.0677	1.39 (0.47 to 4.13)	0.5490	NA	NA
Coronary heart disease (yes vs. no)	8.55 (3.45 to 21.19)	<0.0001	3.78 (1.27 to 11.24)	0.0170	4.18 (1.53 to 11.46)	0.0053
Other heart diseases (yes vs. no)	3.80 (1.39 to 10.40)	0.0095	1.42 (0.45 to 4.48)	0.5467	NA	NA

Abbreviations: AS, ankylosing spondylitis; CI, confidence interval; NA, not applicable.

Of the 21 AS patients who developed stroke, 15 (71.4%) were male. The mean age of these 21 AS patients was 38.0 years (SD = 6.9), elder than that (31.3 years, SD = 7.6) of the remaining 4541 AS patients who did not develop stroke (p<0.0001). The prevalence of diabetes mellitus, hypertension, dyslipidemia, coronary heart disease, and other heart disease of these 21 AS patients at baseline were 0%, 4.8%, 9.5%, 4.8%, and 4.8%, respectively. There was lack of significant difference in the prevalence of the above medical co-morbidities between the AS patients who developed stroke or not.

### Sensitivity analysis

In order to assess the robustness of our results, we carried out the following sensitivity analyses. First, in the present study, the diagnosis of AS was entirely determined by ICD codes from the insurance database, and may be less accurate than those obtained through a standardized procedure. Moreover, the radiographic data are not available in the NHI database. Therefore, we performed additional analysis, using a more rigorous definition that included only AS patients who received two principal diagnosis of AS with at least one being made by a rheumatologist, orthopedist, or physiatrist. The results showed that 3719 (82%) out of the original 4562 patients in the AS group fit this more rigorous case definition. The estimated adjusted HR obtained from this analysis was 1.79 (95% CI, 1.01 to 3.18), consistent with the estimates from the original analysis (Table 2, right column, adjusted HR 1.93, 95% CI, 1.16 to 3.20). Second, while AS patients who had at least 2 ambulatory visits in 2001 were considered for inclusion, the non-AS group consisted of age and gender matched patients who had at least one ambulatory visit in 2001. It may raise a concern that the non-AS group would consist of a potentially healthier population requiring fewer ambulatory visits, which may have had an influence on the risk of stroke. Therefore, we performed additional analysis restricting the non-AS group inclusion criteria to only subjects with at least 2 ambulatory visits in 2001. The majority of the subjects in the non-AS group (21649 out of 22810, 94.9%) had two or more ambulatory visits in 2001. The adjusted HR of ischemic stroke for AS was 1.89 (95% CI, 1.14 to 3.14), which is almost unchanged compared to the original estimates (adjusted HR 1.93, 95% CI, 1.16 to 3.20). Third, as shown in Table 1, the AS group had a higher prevalence of dyslipidemia, coronary heart disease, and other types of heart disease compared to the non-AS group. As these co-morbidities may also contribute to the risk of ischemic stroke, we conducted an analysis excluding all patients (in both the AS and non-AS groups) with any one of the co-morbidities listed in Table 1 (i.e. diabetes mellitus, hypertension, dyslipidemia, coronary heart disease, and other heart disease). The adjusted HR of ischemic stroke for AS was 2.20 (95% CI, 1.25 to 3.90, p = 0.0065), still supporting that AS is associated with a higher risk of developing ischemic stroke. Fourth, we performed a competing risk analysis accounting for death due to causes other than stroke as a competing risk. The adjusted HR of ischemic stroke for AS is 1.84 (95% CI, 1.10 to 3.08), which is very close to the original estimates (adjusted HR 1.93, 95% CI, 1.16 to 3.20).

## Discussion

The major finding in our study was that, in young patients with AS, AS was associated with a 1.9-fold increased risk of ischemic stroke. This association was still seen after controlling for common vascular risk factors. The 2-year ischemic stroke-free survival rate for the AS subjects was lower than that for the non-AS group. Our findings are consistent with two previous observational studies carried out in the US (prevalence ratio: 1.7, 95% CI, 1.3 to 2.3) [9] and in Quebec (prevalence ratio: 1.25, 95% CI, 1.15 to 1.35) [10]. The mechanism responsible for the association between AS and ischemic stroke is unclear; however, we propose the following explanations.

Inflammation plays an important role in the pathogenesis and progression of atherosclerosis [3], [12]–[15]. Previous studies have shown that, compared to patients without AS, AS patients have higher levels of inflammatory markers, such as interleukin 6, tumor necrosis factor alpha, and C-reactive protein [3], [16], [17]. In addition, AS patients have been reported to show early features of atherosclerosis, such as an increase in intima media thickness in the carotid arteries [18]–[22] and impaired flow-mediated dilatation in the brachial arteries [19], [23]. Thus, the increased risk of ischemic stroke in the AS group may result from accelerated atherosclerosis caused by systemic inflammation.

Heart disorders, such as aortic insufficiency, mitral valve disease, and cardiomyopathy, are part of the extraskeletal manifestations of AS [24], [25]. These heart disorders may also contribute to a higher risk of ischemic stroke [26], [27]. However, in our study, although the AS group had a higher prevalence of coronary heart disease and other heart diseases, AS remained an independent risk factor of ischemic stroke after controlling for vascular risk factors and heart diseases in the multivariate analysis. The adjusted hazard ratio of ischemic stroke for the AS group (adjusted HR: 1.93, 95% CI: 1.16 to 3.20) in the multivariate analysis is very close to the crude HR (1.98, 95% CI: 1.20 to 3.29) in the univariate analysis (Table 2). These findings suggest that the increased risk of ischemic stroke in the AS group is independent of the heart involvement in AS.

Non-steroidal anti-inflammatory drugs (NSAIDs) are widely used for treating AS [28], [29]. However, the use of NSAID was not evaluated in our study because observational studies on the effects of NSAID exposure on vascular risks are potentially confounded by indication, as patients with more severe rheumatic diseases are likely to receive higher NSAID doses and also to be at higher disease-related vascular risk. In addition, since NSAIDs are widely available as over the counter medications, their use is not readily assessable in the insurance database. It is therefore difficult to separate the effects of NSAIDs from the biological impacts resulting from AS. Moreover, it remains controversial whether NSAIDs is associated with an increased risk of stroke. A recent large-scale meta-analysis of 280 trials of NSAIDs versus placebo and 474 trials of one NSAID versus another NSAID showed there was no evidence that any NSAID, including selective COX-2 inhibitors and traditional NSAIDs, significantly increased the risk of stroke [30]. Therefore, the use of NSAID was not included in our analysis.

In the present population-based study, the estimated prevalence of AS was 0.12% using case definition that requires at least two ambulatory visits with a principal diagnosis of AS in 2001. This prevalence estimate is relatively lower than that obtained from a community-based survey on the prevalence of rheumatic diseases in Taiwan [31] which used a 2-stage screening process in 1992. In that study, the estimated prevalence of AS in the adult Taiwanese population ranged from 0.19 to 0.54% [31]. Since some AS patients with mild symptoms who did not seek medical service would not be recorded in the NHI database, our study may tend to recruit patients with more severe or active AS, and it can be expected that the estimated prevalence of AS in our study would be lower than that reported from the previous community-based survey.

The strength of the present study was the use of a longitudinal population-based NHI database. The NHI program is a single-payer compulsory social insurance program with considerably high coverage rate in Taiwan. The barrier to medical access is negligible because the NHI system allows patients to visit any clinic or hospital freely without referral by a general practitioner, and patients pay only about $5–$15 USD at each visit. Considering the neurological deficit and functional disability related to stroke, and the minimal barrier to medical access in Taiwan, it can be expected that most patients who developed stroke would seek medical help and would be captured in the NHI database, which enabled us to identify all incident cases of stroke and establish a temporal relationship between AS and ischemic stroke. Nevertheless, several limitations should be acknowledged. First, the diagnoses of AS, ischemic stroke, and medical comorbidities were determined using the ICD codes from the NHI claim database, and there may be concern about the diagnostic accuracy of the database. However, the Bureau of NHI has formed different audit committees that make it a rule to randomly sample the claims data from every hospital and to review charts on a regular basis to verify the diagnostic validity and quality of care. In addition, one validation study that evaluated the validity of the NHI database for patients with a principal diagnosis of ischemic stroke showed that the NHI database appears to be a valid resource for population-based research in ischemic stroke [32]. Accordingly, the NHI claim database is an established research database and independent studies have demonstrated the validity of the data [33]. Furthermore, we performed sensitivity analyses using various case definitions, and found that AS was consistently linked to an increased risk of developing ischemic stroke, suggesting that our results are robust to different case definitions. Second, although we excluded subjects with a previous diagnosis of AS during year 2000 to increase the likelihood of identifying AS patients newly diagnosed in 2001, it was possible that some prevalent AS cases with more longstanding AS who had not sought medical care in 2000 but coded for the first time in 2001 based on our database, were included in the AS group. Third, due to the inherent limitation of the NHI database, information was lacking regarding lifestyle factors, such as smoking, alcohol consumption, and obesity. Moreover, since traditional vascular risk factors, such as diabetes, hypertension and dyslipidemia, are disorders with an insidious onset, some asymptomatic vascular risk factors may not be captured in the NHI database. Therefore, the prevalence of vascular risk factors may be underestimated in both the AS and non-AS groups. These potential confounders may lead to residual confounding and may affect the interpretation of our findings. Fourth, the follow-up time was only 2 years and the long-term effects of AS on the development of ischemic stroke cannot therefore be evaluated. Finally, since Taiwanese are mainly of Chinese ethnicity, it is uncertain whether our findings can be generalized to other ethnic groups.

In conclusion, the present population-based longitudinal follow-up study demonstrates there is an increased risk of ischemic stroke in young patients with AS and highlights the importance of early risk assessment for ischemic stroke in such patients. Further long-term follow-up study would be required to validate our findings and to investigate the underlying pathophysiological mechanism.
